# COVID-19 and suicide: Evidence from Japan

**DOI:** 10.1016/j.lanwpc.2022.100578

**Published:** 2022-09-05

**Authors:** Matthew J. Spittal

**Affiliations:** Melbourne School of Population and Global Health, The University of Melbourne, Melbourne, Australia

Early projections painted a bleak picture of a suicide epidemic following the emergence of the COVID-19. This stemmed from widespread concerns that an unintended consequence of the health mandates designed to limit COVID-19 infections was deteriorating mental health and that this could lead to increases in suicides. This was amplified by poor media reporting^1^ and by early studies forecasting high suicide rates as a consequence of changing health and economic conditions. Yet the evidence to date suggests rising suicide rates have not occurred in most countries. Rather, suicides are generally either lower than what would be expected (based on pre-pandemic trends) or are no different.^2^ The exception to this trend is Japan where suicide rates initially declined by around 14% but then began to rise.^3^ Suicide rates now appear to be higher in Japan than they were pre-pandemic for many age and sex groups.^4^, ^5^, ^6^

Published in *The Lancet Regional Health – Western Pacific*, Goto and colleagues^7^ focus on young people (aged 10-19 years), examining both the timing of changes in suicide rates and possible explanations for changes they observed. Using an interrupted time series design, they show that youth suicide rates began increasing in April 2020 until a peak in September, and then declined. Rates remained slightly elevated between January and April 2021 (when the study ended). Other analyses presented in the study suggest an increase in suicides of between 50% and 86% between August and November 2020 compared with pre-pandemic trends. The authors identified several factors associated with the increase in suicides including family-related concerns, mental illness, social concerns and academic concerns.

No other country has experienced increases, not just in youth suicides but suicides across a range of age and sex groups, like Japan has.^6^ This raises interesting and important questions about why Japan is different from other countries? The authors suggest several factors that may have exacerbated suicides among young people. One possibility is that returning to school in August may have been an additional stressor for students. Another possibility is that because most young people live in a household with other family members, many were exposed to the stressors experienced by other family members. Finally, the authors suggest that young peoples’ lack of access to their wider social circles – necessary to reduce the transmission of COVID-19 – may have impacted on their mental health because of the role social connections play in buffering against many problems. These are plausible hypotheses but do not explain why youth suicides have not risen in other countries where many of the same factors were in play.

In addition to the increased suicides amount young people, other studies have identified increased suicides in Japan amongst employed people, adult women and married adult women who are not in paid employment (at the time of death).^3^^,^^8^ This may reflect the adverse economic effects of the pandemic on industries that employ are large proportion of women – the service, retail and travel industries. Ueda and colleagues^8^ note that in Japan, the number of people employed in non-permanent positions, two-thirds of which are held by women, decreased consecutively in the first 8 months of the pandemic. This is corroborated by monthly survey data during the early phases of the pandemic showing worsening depression and anxiety symptoms among women under the age of 40.^8^ Similarly, the increased risk of suicide among young people observed by Gato and colleagues, and by others,^3^^,^^5^^,^^6^^,^^9^ may reflect the fact that many young people are employed in low-skilled occupations and in precarious employment. Taken together, these findings point to economic and employment vulnerability as important risk factors for suicide at this stage of the pandemic and highlight the need to ameliorate their worst effects. This is particularly important given than many countries are now experiencing low wage growth, high inflation, and rising interest rates – a conflation of events that could lead to many countries experiencing an economic recession.

Given the worsening economic outlook and the fact that the pandemic is likely to continue for some time, it is important to continue to monitor suicide trends in as many countries as possible. Identifying any increases in suicide rates early on will better equip decision-makers to respond with evidence-based interventions quickly and to target high-priority groups. Now is also the time for governments to make greater investments in public mental health, including investments in the prevention of mental disorders, improving mental wellbeing and promoting resilience.^10^ The full effects of the pandemic on suicide are not yet known and continued vigilance is needed to ensure that we avoid the grim outcomes that were forecast at the start of the pandemic.

## Declaration of interests

The author has nothing to declare.
